# Enhancing the public sector’s capacity for inclusive economic participation of disabled youth in rural communities

**DOI:** 10.4102/ajod.v5i1.189

**Published:** 2016-07-22

**Authors:** Lieketseng Ned, Theresa Lorenzo

**Affiliations:** 1 Centre for Rehabilitation Studies, Stellenbosch University, South Africa; 2 Department of Health and Rehabilitation Sciences, University of Cape Town, South Africa

## Abstract

**Background:**

The capacity of service providers in the public sector to deliver inclusive services is essential to implement strategies that will allow the full participation of disabled youth in development opportunities in the rural context.

**Objectives:**

This article sets out to describe the capacity of service providers in facilitating the participation of disabled youth in economic development opportunities.

**Method:**

An instrumental, embedded single case study informed the research design. The sample consisted of five disabled youth, four family members and six service providers. Data was gathered through in depth individual interviews and focus group discussions. Data analysis was done inductively and thematically. In the discussion, the interpretation used organisational capacity elements as a framework.

**Results:**

The findings indicate a perception of disability as a multifaceted and challenging issue with different orientations to service delivery, based on the understanding of the impairment and disability. There is a strong focus on impairment and negative attitudes.

**Discussion:**

An asset-building approach could facilitate awareness of the capacities of disabled youth and thus shift negative attitudes to enabling attitudes. The vague strategies for youth and women reflect an organisational attitude that seems non-committal to its core agenda of inclusive development, which would ensure equal opportunities for participation by disabled youth.

**Conclusion:**

An appreciative process of facilitating a deeper understanding of the needs of disabled youth would assist service providers to reconceptualise disability within an expansive framework of equal opportunities and active citizenship.

## Introduction

Being young and poor in South Africa is already tough. Adding a disability to that equation multiplies the difficulties. Apart from various practical challenges to mobility and accessibility, the prospects disabled youth have for skills development and finding work are often bleak (Cramm, Lorenzo & Nieboer 2013).

The World Disability Report states that disability affects as much as 15% of the world population (WHO 2011). Whilst this figure is highly uncertain and clearly influenced by the prevailing definition of *disability*, it is nevertheless an important indication of the enormity and impact of disability on individuals, families, local communities and societies (Eide, Khupe & Mannan 2014). The day-to-day experiences of disabled youth reinforce their sense of social exclusion and isolation.

It has been found that lower rates of labour market participation are one of the important pathways through which disability may lead to poverty (Braitwaite & Mont 2009; Hoogeveen 2005; Scott & Mete 2008). Across the world, several studies indicate that working-age disabled people still experience significantly lower employment rates and much higher unemployment rates than persons without disabilities (Lorenzo *et al*. 2013; Mitra & Sambamoorthi 2006; Mete 2008). In South Africa, the employment rate is very low. Whilst youth are most affected by minimal employment opportunities (with a 25% unemployment rate in the fourth quarter of 2010; Stats SA 2011), disabled youth – specifically those living in rural areas – suffer the most (with a 12.4% employment rate for disabled people; Stats SA 2011). To address the low rates of employment for disabled people, many countries have laws prohibiting discrimination on the basis of disability, with the aim of improving access to the formal and informal economy and widening social benefits.

### Policy context

The South African government has developed legislation and policies that emphasise participation and inclusion of people with disabilities. Firstly, the South African Constitution identified the need for the integration of disabled persons, thereby acknowledging their rights to employment (Howell, Chalklen & Alberts 2006). Secondly, both the *Employment Equity Act* and the *Skills Development Act of 1998* (Department of Labour 1998a, 1998b) advocate for work opportunities to be created for disabled people. The policies were established in recognition of the discriminatory employment practices that resulted in discrepancies in employment and income in South Africa during the apartheid era. The aim of these acts was to promote the constitutional rights of equality, eliminate unfair discrimination in employment and achieve a diverse workforce broadly representative of the people. For example, the increase in access to employment in the open labour market, development of small, medium and micro-enterprises and entrepreneurship projects are employment strategies that should always be considered (Lorenzo, Van Niekerk & Mdlokolo 2007). Furthermore, disabled youth should have the same access to social security measures as others in the community (Maart *et al*. 2007; UN 2006).

Other policies such as community-based rehabilitation (CBR) guidelines (WHO 2010) and the United Nations Convention on the Rights of Persons with Disabilities (UNCRPD) (UN 2006) promote the participation and inclusion of disabled persons in employment opportunities, particularly in developing countries. Embedded in these policies are the principles of participation and inclusion that should inform service providers when implementing these policies. To include disabled persons into mainstream society, an understanding of the mechanisms contributing to the disadvantaged situations of disabled people globally is needed (Eide *et al*. 2014).

### Barriers to inclusive economic empowerment

Across different provinces in South Africa, the major barriers to employment for disabled youth include poor health, minimal financial resources, inadequate skills and lack of job opportunities (Cramm *et al*. 2013; Grut *et al*. 2009; Lorenzo & Cramm 2012; Lorenzo, Motau & Chappell 2012; Lorenzo *et al*. 2013). These studies also identified minimal provision of mobility technology, communication devices and self-care products; poor retention systems for education and training for skills development to ensure employability; poor dissemination of information and use of communication systems; and inadequate support from family, which was further compounded by the discriminatory attitudes of community and those in authority.

### Capacity of service providers for service delivery

The abilities and competencies of service providers in the public sector to deliver inclusive services are essential to ensure the full participation of disabled youth in the rural context. Although some disabled people are supported by social security (a disability state grant provided by the government in the form of cash transfers), they still have limited economic empowerment opportunities. In seeking to address disabled people’s opportunities for participation, Hess-April (2006) and Philpott (2004) argued that networking and intersectoral collaboration are essential in ensuring successful disability awareness and inclusion and for strengthening referral systems.

Service delivery systems and structures remain fragmented, and available resources have limited impact because many service providers do not realise the need for systematic efforts to adequately prepare disabled persons for participation in the economy (Duncan, Sherry & Watson 2011). Several factors influencing service delivery have been reported, namely, insufficient training and teaching about disability concepts and policy, which would enable district staff to put policy into practice; a poor basis of learning and information from which service providers operate; insufficient knowledge to interpret policy at the level of service provision; limited provincial support and minimal resources; and poor cross-sector collaboration due to limited awareness of official counterparts in each of the sectors (Duncan *et al*. 2011). In addition, some disabled youth have reported being turned away from facilities by practitioners and service providers who are not trained to deal with them, whilst service providers and support services fall short of their needs and often do not take cognisance of policy information (Meyiwa 2010). Saloojee *et al*. (2006) investigated the unmet health, welfare and education needs of disabled children in a poor part of South Africa. Their findings revealed that caregivers have limited money to access services due to distances and inadequate awareness and knowledge of the correct healthcare and available services as major reasons for not utilising these services. Doctors and nurses have narrow awareness of the opportunities for referral to rehabilitation services where disabled youth could gain access to resources to promote their development (Lorenzo & Cramm 2012). These studies indicate that there has not been much change regarding economic inclusion for disabled youth.

#### Justification for the study

Despite government efforts to reintegrate disabled people into the economic environment, Booth and Ainscow (2002) reported that, globally, young adults with disabilities remain excluded from full participation in society and from economic independence. Unemployed disabled people continue to struggle to engage equally and rightfully in society (Howell *et al*. 2006). Due to limited access to employment, disabled youth experience socio-economic exclusion (Maart *et al*. 2007; Lorenzo *et al*. 2013). Moreover, despite the UNCRPD’s positive influence on other international, regional and national policies integrating disability as a human rights issue, there are problems with implementation (Eide *et al*. 2014).

Using Kaplan’s (1999) intangible elements of organisational capacity, this article explores the capacity of service providers within the public sector to facilitate the economic participation of disabled youth in a rural village of Cofimvaba in Eastern Cape, South Africa, as one area needing attention. The intangible elements of capacity (Kaplan 1999) are conceptual understanding; organisational attitudes; and the vision and strategies employed by service providers in promoting the inclusion of disabled youth in economic empowerment opportunities.

## Research methodology

A qualitative case study design was chosen. The case study approach provides a ‘systematic and in depth investigation of a particular instance in its context in order to generate knowledge’ (Rule & John 2011:4), as it enables holistic and meaningful, context-related knowledge and understanding about real-life events (Yin 2009). This tradition of qualitative inquiry was deemed appropriate, as it allowed the participants to be bounded as cases whilst situating each group in its historical, political, economic, as well as socio-cultural contexts, demanding multiple sources of data (Stake 2008). The type of single case study adopted was an instrumental, embedded single case study approach (Stake 2005), which has more than one unit or subunit of analysis. In this study, the service providers from the Departments of Education, Agriculture, Health and Social Development and Local Government (the municipality), disabled youth and their families within Cofimvaba, Intsika Yethu Municipality, were bounded as a single case to explore how the service providers facilitated the participation of disabled youth in economic opportunities.

### Setting

Cofimvaba is a rural area in the Intsika Yethu Municipality in the Eastern Cape, South Africa. The village is 79 km east of Queenstown on the route to Butterworth, in Thembuland. It has a high rate of unemployment. The district is comparatively poor, with 75% of its people living below the poverty line (Department of Provincial and Local Government 2003). The recent census (Statistics South Africa 2011) reported that there were 8783 disabled people (with various forms of impairment) in Cofimvaba, constituting 7.6% of the total population. Only 11% of disabled youth are employed and earning a salary, with approximately 62% considered as economically inactive (Statistics South Africa 2011). All the service providers are located in the town of Cofimvaba. Each service provider is allocated a surrounding village and is responsible for supporting and assessing the needs of the village and monitoring any projects initiated and supported by various organisations. Service providers have to travel distances on gravel roads to access the communities using government transport.

### Sample

Purposive sampling was used to select service providers, for their knowledge and insight of service delivery in this community, and disabled youth and their families as the users and beneficiaries of these services (Rule & John 2011). This article focuses on the sample, which consisted of six service providers, five disabled youth and four family members. The service providers, all with two or more years’ experience, included three community workers, one Social Development practitioner, one health professional and one special unit manager of the local municipality. The findings on disabled youth and their families will be reported to communicate their perspectives of the capacity of service providers at ground level, as they are the beneficiaries of the services delivered by these service providers. The local councillors were invited but were not available to give their perspectives.

Initially, disabled youth were contacted from a database of disabled youth compiled from a phase 1 survey of disabled youth in rural areas (DYRA) carried out in Cofimvaba in 2011. Due to difficulties locating most of the youth in the community, snowballing was then used: the two disabled youth located referred the researcher to other disabled youth they knew within the community. Each disabled youth (two females and three males) then came with a family member (two mothers, one grandmother and one father) who volunteered to be part of the study. All disabled youth and family members were unemployed community members from one of the villages in Cofimvaba. Although the inclusion criteria of disabled youth was intended to permit examination of both employed and unemployed disabled youth in order to draw an analysis of barriers and facilitators from the success stories of employed youth and the stories of those who could not find working opportunities, those who were already working had moved out of Cofimvaba. Moreover, the majority of disabled youth had either an intellectual or sensory disability, implying that youth with mobility and psychosocial disabilities may be more transient, moving to other cities to look for employment.

### Data generation methods

Multiple data generation methods were utilised to develop a thorough understanding of the case (Yin 2003). This article reports data from the individual in depth interviews with service providers, focus groups interviews with the disabled youth and their families, and a reflective journal that was kept throughout the research process by the lead author as part of her postgraduate qualification in Disability Studies. The data generation process took approximately 3 months. The focus groups with disabled youth and family members took place in a community hall. A set of guiding questions were used to initiate the discussion. The interaction in the group allowed for observation of similarities and differences between families’ opinions and experiences (Morgan 1997) in supporting their disabled daughters and sons in accessing development opportunities, as well as for understanding the support that they, as the families of disabled youth, required (and got) from service providers. The service providers chose individual interviews in preference to focus group discussions and were each interviewed in their own workplaces. The data gathered from these individual interviews were validated in follow-up meetings as well as in the combined member checking meeting, where all service providers, disabled youth and their families came together for member checking of findings.

**FIGURE 1:**
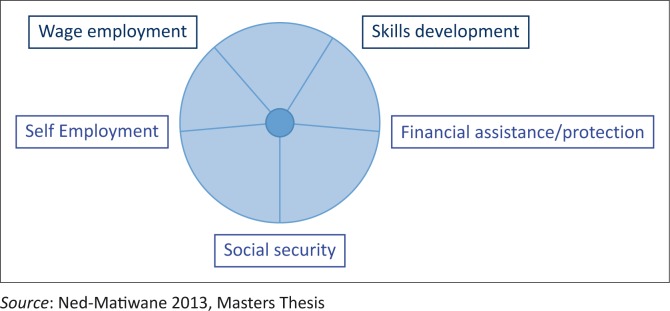
Wheel of Opportunities for Participation.

As a tool to trigger information-gathering in the focus groups and in the individual in depth interviews, the Wheel of Opportunities for Participation (WOOP) incorporated the elements of the Livelihood component from the CBR guidelines, namely, skills development, self-employment, wage employment, financial services and social protection (WHO 2010). In the interviews, the data gathered related to the service providers’ skills, knowledge, attitudes and approaches for facilitating the participation of disabled youth in economic opportunities linked to the five elements of the Livelihood component. The interviews began with an open-ended question, and the participant’s response guided the researcher to further questions. In the focus groups, the data gathered related to the disabled youth’s participation in the opportunities (from the perspectives of the disabled youth and their families) and outlining the barriers and facilitators in relation to the services provided. They also reflected on the support they required from the service providers and their awareness of the current services (see Figure 1).

All participants were asked to plot on the WOOP their perceived level of participation of the disabled youth for each element. They then gave reasons for plotting whether the participation was high, low or average and identified strategies for improving the low participation to high.

All the participants gave permission for the interviews to be digitally recorded. The interviews were conducted in English for the service providers and isiXhosa for the disabled youth and their families. All focus groups and interviews were 1.5 hours long, including rest breaks.

A reflective journal was kept after each meeting with all the participants to record any critical incidences, as well as the lead author’s thoughts, experiences and learnings throughout the research process, especially the methodological aspects of the research. This tool was a useful reference point during the data analysis as it contributed to the interpretation of data and development of an argument.

### Data analysis

The analysis explored the understanding of the service providers, disabled youth and their families regarding disability and economic opportunities. It identified their visions and strategies, as well as challenges they experienced in developing disability-inclusive economic opportunities for disabled youth. These elements are more subtle and less easily quantifiable concepts, which are first and foremost critical when looking at organisational capacity (Kaplan 1999). The perceptions of the service providers were explored alongside the perceptions of the disabled youth and families, which contributed significantly to comparing their perceptions on the skills and abilities of the public sector to support disabled youth, as well as what was happening at ground level.

All isiXhosa interviews were translated and transcribed simultaneously and verbatim. They employed thematic coding: firstly, the primary researcher familiarised herself with the data, to understand the overall meaning of the information (Babbie & Mouton 2001). Then a preliminary analysis of each transcript was done, to identify codes that emerged from the raw data, which were grouped together to form categories. These categories were grouped, in turn, to form themes. A second level of analysis was done inductively across the different data sources, to verify categories and themes, until data saturation was reached.

To ensure rigour and trustworthiness of the data analysis and interpretation processes, we followed up with the participants of both groups (service providers, disabled youth and their families) together for member checking of themes and categories (Creswell 1994).

Ethical approval for this study was obtained from the Faculty of Health Sciences Human Ethics Research Committee at the University of Cape Town. In the next section, pseudonyms are used to protect the anonymity and confidentiality of the participants.

## Results

Two themes emerged related to the service providers’ capacity within the public sector to facilitate inclusive economic opportunities, namely 1) generating an understanding of disability, and 2) the competing visions and strategies. The views of the service providers are compared with those of the disabled youth and their families.

### Generating an understanding of disability

Different understandings of disability by service providers revealed an impairment focus, which was multifaceted, coupled with a continuum of attitudes. The capability to participate was based on the type of impairment, with significantly more awareness of physical impairments and less of sensory or intellectual impairments. There was minimal recognition of the role of attitudinal, personal and environmental factors in the insufficient engagement and inclusion of disabled people. Different views were shared by the services providers:

‘To be disabled does not mean that you cannot do ‘ABC’ … If you just remove those barriers to show what these people are capable.’ (Bonang, Local Municipality)

‘A person who has been injured, and [*it has*] affected a specific limb … Some of them have the ability – like the paraplegics, because they can use their hands – but the quads cannot be employed.’ (Beauty, Health)

‘There are certain things they are not able to do because they struggle a lot … Not to say he or she cannot do anything for herself or himself … Our programmes include people who are basically coping, and you find out that they’re very good with handwork or hard labour; but you cannot put them in charge as part of the executive, as sometimes they lose it … I think even, not ‘physical’ per se, I think even mental; what I can say is that mostly we see physically disabled people, because we can relate.’ (Cebo, Social Development)

The disabled youth and their families felt that, in their context, they came across as people who were unable to do things and become productive and contributing members of the community. Some disabled youth stated:

‘As people who are disabled, we are looked down upon, and only used for purposes of getting votes when it is voting time.’ (Buhle, disabled youth)

‘I think that we come across as people who are unable to work productively, and we end up believing that. For instance, I once lost a job and I was just told that I will be phoned, but till today, that was a lie. It was because of my disability.’ (Onke, disabled youth)

The parents of disabled youth experienced discrimination and the negative attitudes of service providers as limitations of their capacity to support disabled youth from accessing skills development opportunities essential for work:

‘They are able to do handwork, but these skills need to be enhanced through further education and training, which is what we do not have here. They are discriminated against in schools and excluded from available opportunities.’ (Nosiphiwo, mother)

The attitudes of the service providers revealed that they recognised ability and productivity amongst disabled youth, but they also held stereotypes of disabled youth’s dependency on grants. These perceptions influenced how the service providers facilitated inclusion. They fluctuated between different views of perceiving disabled youth as being very positive and believing in their potential to doubting and blaming them for their exclusion. Table 1 reflects the continuum of both disabling and enabling attitudes quoted from the service providers.

**TABLE 1:** Continuum of attitudes.

Disabling attitudes	Enabling attitudes
‘I do feel that the grant has a negative factor, because they just get satisfied with the grant and [*do*] not think that they can do something else that will assist them [*to*] sustain their living.’ (Beauty, Health) ‘They are capable of being employed … but our people are now taken by this social security – which is the disability grant – so when the person gets a disability grant, they take the back seat because they know that “I have something that I am getting at the end of the month”.’ (Cebo, Social Development) ‘It is about people who are interested, so if you are, you can join also.’ (Beauty, Health)	‘They are the most productive.’ (Cebo, Social Development) ‘Like any other normal human being in the community, there are members who are active and those who are passive.’ ‘Some are participating; they are very active. You must not undermine them; there are those who are highly active.’ (Bonang, local municipality) ‘I think we need to conduct a lot of workshops [*for*] the people in terms of why it is important for them to participate in their phase of government. Generally it is average to the whole public, not specifically to them, because I treat them equal to the other people in this question of participation; they are also not left out.’ (Bonang, local municipality)

*Source*: Ned-Matiwane 2013, Masters Thesis

### Competing visions and strategies

The service providers identified various visions and certain strategies for facilitating economic inclusion for the disabled youth. These ideas are reported in relation to those of the disabled youth and their families as well as their challenges. The categories *being able* and *fighting poverty* were the identified vision.

*Being able* to gain confidence, self-reliance, resilience, skills, as well as *fighting poverty*, were identified as contributing factors in promoting inclusion:

‘We are not just going to people and assisting them; there should be something that they are already doing. Then they can come and apply for funding… [*We*] would like to see them believing in themselves, and being able to do things to gain that self-reliance and resilience; because you will find out that mostly, disabled people, they do not believe in themselves… The main objective of Social Development is to fight poverty, more especially in a sustainable project. We only fund projects that will assist communities – for instance, farming.’ (Cebo, Social Development)

‘Here at Social Development, we fund youth and women. We cover that they create their own businesses, which is self-employment. Jobs are created in that they are given the money to work for themselves, so initiating the project and running it is a job, and they get money from the project, which is the earnings.’ (Cebo, Social Development)

‘In Siyazondla *[‘We are nourishing’*] they have gardens in their households – this is only to provide for themselves at home. Siyakhula [‘We are developing’] is like an expansion of that, to producing more to sell.’ (Akhona, Agriculture)

‘As a Municipality we have as a starting point selected one person per ward to build ramps in their households to those people who are wheelchair bound. To educate people, that after building your rondavel or a flat, do not just build the steps. There must be ramps even for people with disabilities, because we are not living alone, amongst family members or neighbours; we have got people who are wheelchair-bound.’ (Bonang, Municipality)

The categories *it is for youth in general, active in decision making* and *we move together* were identified as the strategies of the service providers to achieve their vision for disabled youth:

‘We just provide service to everyone; there is nothing specific to disabled youth. And also, part of what we do, [it] does not emphasise involving disabled people to ensure that they participate.’ (Cebo, Social Development)

‘There is no special treatment here which is specially designated for … although we need to absorb many people with disabilities; we have to be very careful to not discriminate. So I think some do require some degrees or diploma and some particular certificate.’ (Bonang, Municipality)

‘The type of approach we are using is to collect information from the people in terms of what they exactly want; and as the government of the people, we do exactly what the people really want.’ (Bonang, Municipality)

In comparing these views, disabled youth and their families felt that they wanted to strive for themselves and be able to achieve ordinary aspirations but also recognised that service providers could be more supportive, advising and guiding them in this process:

‘They need to be motivated to strive for themselves…’ (Nosakhile, mother)

‘There is no one to even advise us on how to start those initiatives; there is no one to advise us and enable us to succeed. There is a project here, but we do not know who is helping them, we just saw a project going on. We need people to advise and train us and that is what we need to empower ourselves as the families of disabled children. We need to be assisted.’ (Bobisa, father)

They also wanted to be economically self-empowered:

‘It is mainly the grant that is available here in Cofimvaba, which is what helps people live.’ (Ayanda, disabled youth)

‘We usually borrow money from other community members and return it on pay day … Maybe if I had some capital, I could only take some money from the grants and then start a business … My wish is for them [disabled daughter and son] to be assisted by government to get bursaries for schooling.’ (Nosakhile, mother)

‘I do not know where and how to get further assistance towards capital … We do not know about any other places that help financially for us to start businesses; no one has ever told us about that, which is why we do not know. We have not seen such places … we have not received any help or advice from there apart from the grant [*clinics and hospitals*].’ (Nosakhile, mother)

Family members and disabled youth further shared some strategies for service providers to enable disabled youth to become economically self-empowered:

‘…find help and look for opportunities, and even open opportunities for themselves so that they can stay in their communities and develop.’ (Nosakhile, mother)

‘There need to be people who specifically come to help and train youth on how to manage and start specific initiatives, so that the youth can carry on without outside help to develop them.’ (Nosakhile, mother)

‘They must come to us and discuss with us, so that they can understand us better and our needs; in this way we can learn how to work together and empower each other.’ (Onke, disabled youth)

## Discussion

It is evident from our findings that the understanding of disability still remains predominantly impairment-focused; often the functional capabilities and limitations of disabled youth are perceived to be a result of (and dependent on) the different types of impairment, rather than attitudinal and environmental factors. Duncan *et al*.’s (2011) instrumental case study on service providers from the Departments of Health, Education and Social Development in the Eastern Cape’s Alfred Nzo District found similar results. Kaplan’s (1999) framework states that for an organisation to have capacity, it needs to have an understanding of its world and its role in it. Within the context of the current study, this would ensure a common understanding of inclusive development as well as inform roles and strategies in meeting the development needs of disabled youth in this community. The findings highlight that although the represented departments aim at facilitating inclusive development of all people, they remain vague in their strategies to make this inclusive development a reality. This finding links to organisational intent; all participants showed awareness of having to include disabled youth, yet were not very knowledgeable about disability inclusion and the related policies, as well as aligning these processes with their work.

There is a need for service providers to take a holistic approach, taking into account all aspects of a person’s lived experiences and their context, such as the individual needs related to their impairment, personal and environmental factors, as well as activity and participation restrictions that may impinge on their rights as active citizens. Being inclusive then refers to ensuring that the voice of each person is part of the process of change.

This study revealed that there is a difference between what service providers say they do and what they actually do related to the economic and social inclusion of disabled youth. This difference shows a clear disjuncture between the policy aspirations and the reality of minimal implementation. Dube (2006) identified various reasons for poor implementation of disability-related policy at different levels of government, namely, limited conceptual understanding, poor championship, inadequate arrangements and general lack of capacity. It seems that programme managers do not have the capacity (conceptual understanding, which informs the attitudes and strategies) to mobilise disabled youth to participate in new or existing programmes nor a comprehensive understanding of how to monitor their economic and social inclusion, despite their understanding policy aspirations. The latter speaks to organisational attitude, which is an intangible element of capacity.

Our study further reveals that intellectually disabled youth were viewed as the most productive, particularly in handwork, but not in executive or managerial work. Physically disabled youth were perceived as unemployable, particularly those who were quadriplegic. Kumurenzi (2011) recorded similar findings in the Western Cape, where many disability and rehabilitation services seem to operate using an individual, impairment approach.

Some service providers in this study perceived disabled youth as not wanting to engage in community projects. The perception reinforces the individual model of disability, which states that disabled people are weak, helpless and dependent on charity or professionals, needing care all the time (Terzi 2004). Any restriction of activity or social disadvantage that the individual confronts is deemed to be the inevitable consequence of the impairment (Thomas 2002, cited in Hammell 2006). This perception was often the reason service providers did not mobilise disabled youth, thereby limiting participation in gaining skills. The impact of attitudinal barriers was also observed in a study on people with mental illness who experienced employment discrimination (Stuart 2006). Stuart argued that attitudinal barriers impact significantly on the participation of disabled people in the open labour market. Similarly, Johannsmeier (2007), Lorenzo *et al*. (2013) and Cramm *et al*. (2012) found that negative attitudes of teachers or parents may prevent a child from attending school, and the resulting lack of education will in turn affect employment prospects. This finding confirms the argument of Eide *et al*. (2014) that a key to knowledge production necessary for disability-inclusive developments is a critical view of the dominant understandings of disability and development.

It was shown that disabled youth and their families perceived disability as inability. This may be because the prevailing discriminatory attitudes, marginalisation and experiences of despair had led to occupational disengagement – the absence of engagement in occupations due to a loss of the meaning derived from previously enjoyed occupations (Krupa *et al*. 2009). Drawing from Occupational Science, *occupations* refers to the ordinary, everyday things that people do to meet their various needs, interests and aspirations (Watson & Fourie 2004). Exclusion on the basis of disability has had a profound effect on the self-esteem and confidence of disabled youth and their families, making them think they are incapable and incompetent.

Disabled youth and their families have positive perceptions of disability grants; they view grants as positive enablers for entrepreneurship opportunities, as they provide seed funding for starting small businesses. Similar findings were identified in studies conducted in informal settlements in Cape Town that revealed links between the disability grant and level of participation (Lorenzo *et al*. 2007; Van Niekerk, Lorenzo & Mdlokolo 2006).

Service providers need to understand disabled youth’s choices and that decisions are shaped by the many factors that influence their lives. It is essential that service providers link their strategies for economic inclusion to what is perceived as important and needed by disabled youth and their families. This linkage can be achieved through providing support for and establishing relationships with the people benefiting from these services. It is not a matter of the individual person changing or adapting, but rather a need to address physical inaccessibility, attitudinal and informational barriers in communities through local service provision at the municipal level (Grut *et al*. 2012). Community development workers have been identified as well positioned to facilitate these shifts (Lorenzo, van Pletzen & Booyens 2015).

This enablement approach could be a catalyst for empowerment, so that disabled youth could become agents of their own change. The extension officers, community development workers and local government structures in Cofimvaba need to play this role and be equipped with skills related to mobilising, organising and supporting disabled youth and their families in the planning and implementation of self-help projects (Ned-Matiwane 2013). Seeing disabled youth engaged in productive work has been shown to increase public awareness of youth as capable and equally contributing members of society (Lorenzo *et al*. 2007; van Pletzen, Booyens & Lorenzo 2014).

Shaw *et al*. (2007) asserted that a supportive social context of team effort and meaningful partnership between service providers and disabled people is important to encourage equitable participation for disabled people. Ramphele (2008) suggested that all South Africans need to learn to work together. A feasible attitude-changing strategy would be to engage service providers in transformative processes to help heal divisions (Ramphele 2012). Transformative leadership is one approach to lead a change towards teamwork and collaboration, rather than viewing each other as competitors. This approach is particularly applicable to creating leadership for public service delivery for everyone in rural areas, including disabled youth and their families. Additionally, an asset-building approach could facilitate awareness of the capacities of disabled youth and thus shift negative attitudes to an enabling attitude that realises that disabled youth do have something to contribute, as they also have skills and abilities to contribute to community development (McKnight & Kretzmann 1996). This approach will also lay the foundation for service providers to recognise the skills that are lying dormant at the community level. It would assist with building collaboration and partnerships for community development.

## Conclusion

The CBR guidelines (WHO 2010) presented at the beginning of this article provide a critical framework and strategy for the adoption of disability-inclusive rural community development by all service providers and development workers. This strategy is intended to foster the participation of disabled youth and their families; to challenge existing inequalities, group identities and differences; to raise the self-esteem of currently devalued groups; and to build the confidence to act. It is essential that the service providers and development workers link the activities of the community to priorities for disabled youth.

Whilst many factors contribute to the gaps in integrating disabled youth, the capacity of service providers in rural areas needs to be strengthened to significantly impact on creating real service delivery outputs, in order to realise a truly inclusive society. The following are three recommendations for shifting the thinking about disability and encouraging a two-pronged approach to the social and economic inclusion of disabled youth by service providers:

1. Dialogue must happen between service providers and disabled youth and their families to recognise people’s potential and encourage the reciprocity of ideas related to building consensus on the visions and strategies to promote the social and economic inclusion of disabled youth.
2. Further training and workshops on monitoring disability inclusion are needed to promote a broader understanding of and sensitisation to disability, as well as an understanding of mainstreaming disability.
3. Inclusion of disability issues into government strategies for education, skills development and employment should be addressed systematically.

A holistic understanding of the human rights needs of disabled people is necessary to assist service providers in recognising impairment and functional needs, whilst also being aware of the personal and environmental factors that could be both barriers and facilitators to meeting these needs. This focus will reconceptualise disability within an expansive framework, instead of the narrow views evident in this study, and may contribute to reducing injustices and exclusion based on disability discrimination.
